# The Effect of High Concentration and Small Size of Nanodiamonds on the Strength of Interface and Fracture Properties in Epoxy Nanocomposite

**DOI:** 10.3390/ma9070507

**Published:** 2016-06-23

**Authors:** Yasir A. Haleem, Pin Song, Daobin Liu, Changda Wang, Wei Gan, Muhammad Farooq Saleem, Li Song

**Affiliations:** 1 National Synchrotron Radiation Laboratory, CAS Center for Excellence in Nanoscience, University of Science and Technology of China, Hefei 230029, Anhui, China; hiyasir@mail.ustc.edu.cn (Y.A.H.); ldbin@mail.ustc.edu.cn (D.L.); wchda@mail.ustc.edu.cn (C.W.); gwutopia@mail.ustc.edu.cn (W.G.); farooq@mail.ustc.edu.cn (M.F.S.); 2 School of Chemistry and Chemical Engineering, Hefei University of Technology, Hefei 230029, Anhui, China; psong@mail.hfut.edu.cn

**Keywords:** nanodiamond, epoxy, nanocomposites, interface, fracture properties

## Abstract

The concentration and small size of nanodiamonds (NDs) plays a crucial role in the mechanical performance of epoxy-based nanocomposites by modifying the interface strength. Herein, we systemically analyzed the relation between the high concentration and small size of ND and the fracture properties of its epoxy-based nanocomposites. It was observed that there is a two-fold increase in fracture toughness and a three-fold increase in fracture energy. Rationally, functionalized-NDs (F-NDs) showed a much better performance for the nanocomposite than pristine NDs (P-NDs) because of additional functional groups on its surface. The F-ND/epoxy nanocomposites exhibited rougher surface in contrast with the P-ND/epoxy, indicating the presence of a strong interface. We found that the interfaces in F-ND/epoxy nanocomposites at high concentrations of NDs overlap by making a web, which can efficiently hinder further crack propagation. In addition, the de-bonding in P-ND/epoxy nanocomposites occurred at the interface with the appearance of plastic voids or semi-naked particles, whereas the de-bonding for F-ND/epoxy nanocomposites happened within the epoxy molecular network instead of the interface. Because of the strong interface in F-ND/epoxy nanocomposites, at high concentrations the de-bonding within the epoxy molecular network may lead to subsequent cracks, parallel to the parent crack, via crack splitting which results in a fiber-like structure on the fracture surface. The plastic void growth, crack deflection and subsequent crack growth were correlated to higher values of fracture toughness and fracture energy in F-ND/epoxy nanocomposites.

## 1. Introduction

Epoxy resins are thermosetting polymers mostly used as adhesives [1]. The advantage of epoxy resins is that they are in liquid form at room temperature and can be transformed into rigid form by adding a suitable hardener and heating them for a while. The curing of epoxy resins makes them highly cross-linked materials. The epoxy/hardener ratio and the curing temperature strongly affect the mechanical properties of epoxy resins [2]. Pure epoxies are electrically insulating and mechanically brittle but their properties can be changed by suitable additives. Lee et al. [3] reported the improvements in the micro-mechanical behavior of self-healing epoxy and hardener-loaded epoxy micro-capsules whereas Ramos et al. [4] studied the interphases in epoxy/amine thermosetting systems modified with thermoplastics using the nano-indentation technique. The selection of appropriate filler strongly affects the properties. This selection can be made on the basis of shape, size, and the nature of the filler. Nano-sized fillers have great impact on the mechanical properties of epoxy resins. Lam et al. [5] investigated the enhancement in mechanical properties of epoxy because of the effect of distribution of nano-clay platelets as reinforcements. The interaction between the filler and epoxy network is a key for better mechanical properties. Shukla et al. [6] reported the effect of volume fraction and functionalization of alumina nano-platelets on the mechanical and fracture properties of epoxy while Zhao et al. [1] observed significant improvements in the ductility and modulus of epoxy by using alumina nano-particles as a filler. Kinloch et al. [7] reported a substantial increase in the toughness of epoxy polymer because of silica nano-particles and rubber particles. Furthermore, carbon nano-structures are potential candidates as filler and, in the past, several carbon structures have been used to enhance the mechanical properties of epoxy resins. These carbon nano-structures include carbon nano-fiber, carbon nano-tubes (CNTs), graphene, and nanodiamonds, etc. Sanchez et al. [8] measured the enhancement of the elastic modulus of epoxy using 5 weight percent (wt %) of carbon nano-fiber as a filler. Wang et al. [9] observed an almost eightfold increase in the elastic modulus of epoxy with just 0.5 wt % loading of functionalized CNTs. Wang et al. [10] showed improvements in toughness by the incorporation of functionalized graphene (GO) sheets in an epoxy network. Nanodiamonds (NDs), because of their good thermal and mechanical properties, are a strong candidate as one of the promising fillers. The cluster size of ND particles is in the range of 100–500 nm [11]. The large surface-to-volume ratio gives more space to attach functional groups on the surface of ND. These functional groups effectively changed the mechanical properties of the epoxy resin [12,13]. Sobia et al. [14] and Zhai et al. [15] investigated the effect of low content of NDs on the mechanical properties of epoxy whereas Neitzel et al. [16] studied mechanical properties using nano-indentation with a high content of NDs. In our previous work [11], we also investigated the tensile properties of epoxy-based nanocomposites using a high concentration of NDs. In this work, we have investigated the crack- and fracture-related properties of epoxy-based nanocomposites with NDs as reinforcement.

## 2. Materials and Methods

**Figure 1:**
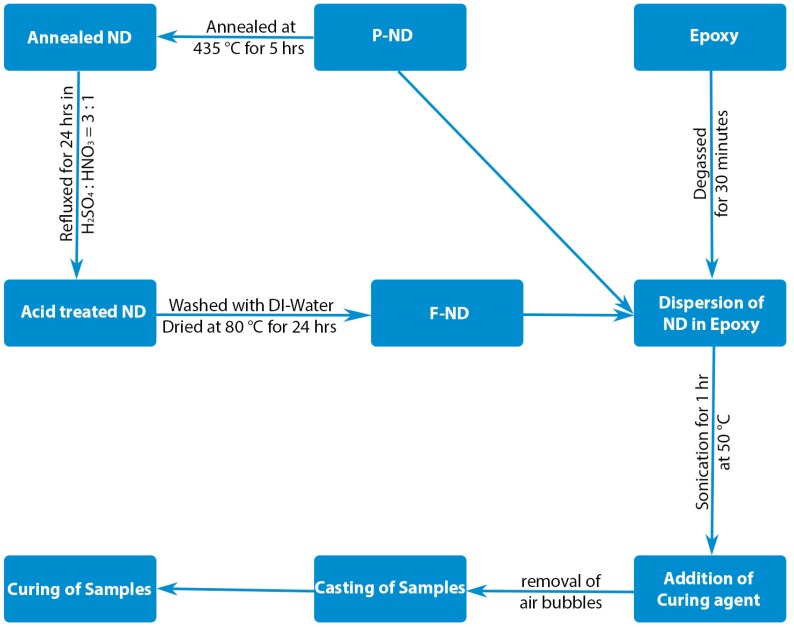
Schematic of nanocomposite formation.

The phase purity of pristine NDs (P-NDs) was about 50% and it was purchased from Henan Union Abrasives Corporation, Zhengzhou, China. As per the supplier’s specifications, the size of the P-ND cluster was less than 500 nm and its color was black. Impurities were removed by annealing the P-ND at 435 °C for 5 h [12,13]. Usually, non-diamond content, i.e., amorphous and graphitic carbon and traces of metals, are considered as impurities [17]. After annealing, P-NDs were treated with a mixture of sulphuric acid (Sinopharm, Shanghai, China) and nitric acid (Sinopharm, Shanghai, China) by using reflex assembly as described elsewhere [18]. During the acid treatment of P-NDs the reflux temperature was kept around 280 °C, followed by continuous stirring for 24 h [11]. NDs were separated from acids mixture by using the centrifuge machine (Analytical Instruments Co., Shanghai, China) and afterwards washed with de-ionized water (Anting Scientific Instrument Factory, Shanghai, China) to neutralize the pH. These washed NDs were termed as functionalized NDs (F-NDs) and in the end were dried at 80 °C for 24 h to get the F-NDs in powder form [11]. Hydroxyl and carboxyl functional groups were attached on the NDs’ surface during the reflux process and the size of F-ND clusters are found to be approximately around 100 nm [11]. The P-ND and F-ND powders were characterized by X-ray diffraction (XRD), Fourier transform infrared (FTIR) spectroscopy, and X-ray photoelectron spectroscopy (XPS). XRD was done on Philips X’Pert Pro Super X-ray diffractometer (PANalytical, Almelo, The Netherlands) with Cu Kα (λ = 1.54178 Å) radiation source, FTIR spectra were acquired using Thermal Fisher Nicolet 8700 (Thermo Fisher Scientific Inc., Waltham, MA, USA) with a resolution of 0.1 cm^−1^ while Thermal Fisher ESCALAB250Xi spectrometer (Thermo Fisher Scientific Inc., Waltham, MA, USA) equipped with an Al anode (Al K_α_ = 1486.7 eV) was used for XPS. The nanocomposites were formed by using P-ND and F-ND as filler while epoxy as a matrix. Commercially purchased epoxy diglycidyl ether of bisphenol-A (DGEBA) (ZhongkKe Scientific, Beijing, China) is used as matrix material while methylhexahydrophthalic anhydride (MHHPA) (ZhongkKe Scientific, Beijing, China) is used as a curing agent. The curing agent to epoxy ratio was 92:100 in grams and this ratio gives the best mechanical properties according to the literature [19]. Predetermined amount of NDs was added to epoxy via sonication for 1 h at 50 °C after degassing the epoxy for 30 min. Temperature-dependent viscosity of epoxy resin helps direct incorporation of NDs. By using mechanical mixing machine (Fluko, Shanghai, China) MHHPA was mixed with the mixture of ND/epoxy for 2 min. Afterwards, the air bubbles inside the mixture were removed by putting it in vacuum for 1 min and then casted into the molds. Standard curing process was used for curing the nanocomposite samples [20]. The schematic of nanocomposite formation is shown in Figure 1. The nanocomposite samples with different concentrations of NDs were prepared and these concentrations are 1, 10, and 20 by weight percent (wt %) of epoxy. The nanocomposite samples were tested according to ASTM D-5045 standard [21] and fracture surfaces were examined by optical microscope and scanning electron microscopy (SEM). Testing, for fracture properties, was done on MTS SANS CMT6503 (MTS, Shanghai, China) with a maximum force of 5 kN at ambient conditions. Optical images were taken on JSZ6D SE2200 microscope (Olympus, Tokyo, Japan) using a 10 W LED lamp and SEM images were taken on JEOL JSM-6700F SEM (JEOL, Tokyo, Japan) at 10 kV.

## 3. Experimental Results and Discussion

**Figure 2:**
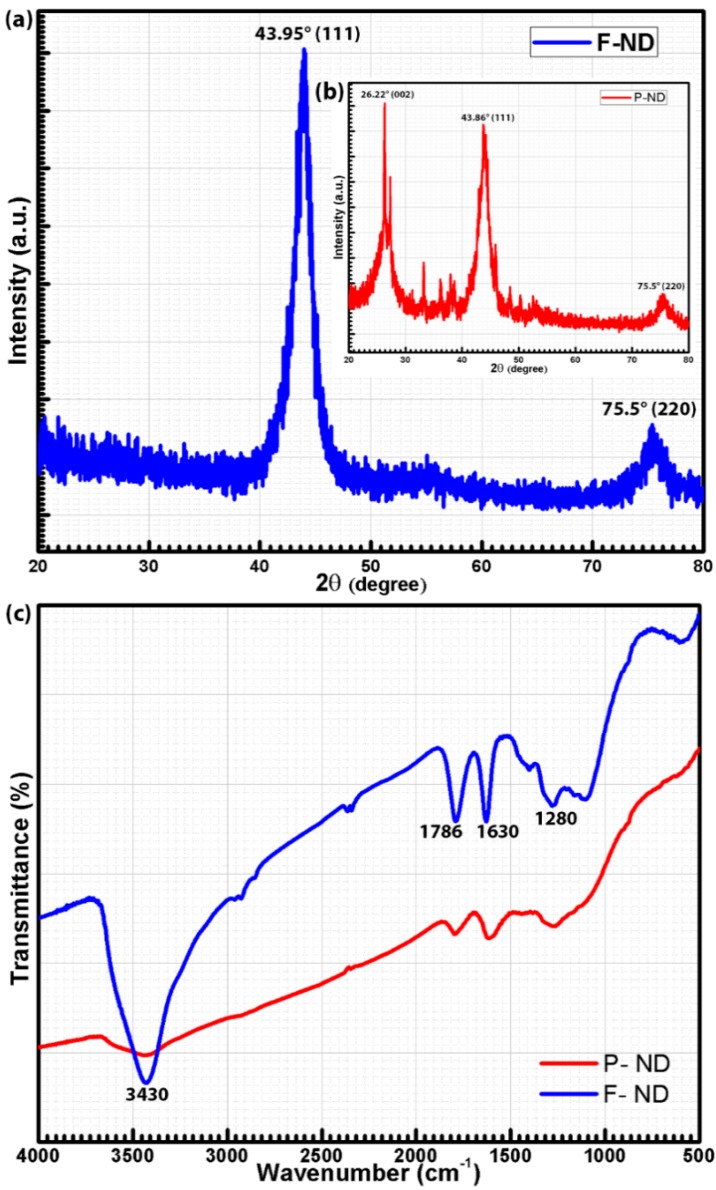
(**a**) XRD pattern of F-NDs and (**b**) P-NDs; (**c**) comparison of FTIR spectra of P-NDs and F-NDs.

The XRD pattern of F-NDs was acquired in the 2θ range of 20°–80° by using the CuKα_1_ radiation source and is shown in Figure 2a,b (inset). The diffraction peaks (see Figure 2a) at 43.95° and 75.5° (JCPDS 03-065-6329 [22]) are attributed to the diffraction planes (111) and (220) of cubic diamond crystals, respectively [17,23]. In Figure 2b, the same peaks are present at 43.86° and 75.5°, respectively, with an additional peak at 26.22° which represents the (002) diffraction plane for graphitic carbon (sp^2^ carbon). The XRD patterns emphasized the dominance of sp^3^ carbon over sp^2^ carbon after the acid functionalization of P-NDs. No amorphous graphite (sp^2^ carbon) peak was observed in the F-NDs. The amorphous graphite shell is present around the ND core which becomes very thin during the functionalization process because of acid erosion [11]. This thin graphite shell cannot be easily detected by XRD [22]. Therefore, the absence of the amorphous graphite peak in the XRD pattern of F-NDs cannot rule out the presence of sp^2^ carbon. It might be there as an impurity and may be undetectable due to the thin shell. In our previous work we had done X-ray absorption near edge spectroscopy (XANES) which is a more sophisticated technique and it showed the presence of sp^2^ carbon in the F-ND sample [11]. The full width at half maximum (FWHM) of the XRD pattern of F-NDs helps to estimate the particle size of the NDs using the Scherrer equation [24] and it is found to be around 4 nm. The FTIR spectra of P-NDs and F-NDs are shown in Figure 2c. According to these spectra, P-NDs have broad and small peaks, but on the contrary, F-NDs have sharp peaks. The peak around 3430 cm^−1^ is a characteristic peak of the stretching vibrations of the OH group. The bending vibrations of the OH groups have a peak around 1630 cm^−1^ [25]. The peak around 1786 cm^−1^ is assigned to the stretching vibrations of C=O for the –COOH group [26]. The peak around 1280 cm^−1^ can be assigned to the stretching vibrations of the C–O group [27]. The peak height and sharpness cannot be a direct measure of the content present, but the relative comparison of peak heights in both spectra gives an idea about the amount of content. It is clearly observed in Figure 2c that the F-ND spectrum has sharper peaks than that of the P-ND spectrum, which is a strong indication of the attachment of carboxyl and hydroxyl functional groups on the ND surface during the functionalization process.

**Figure 3:**
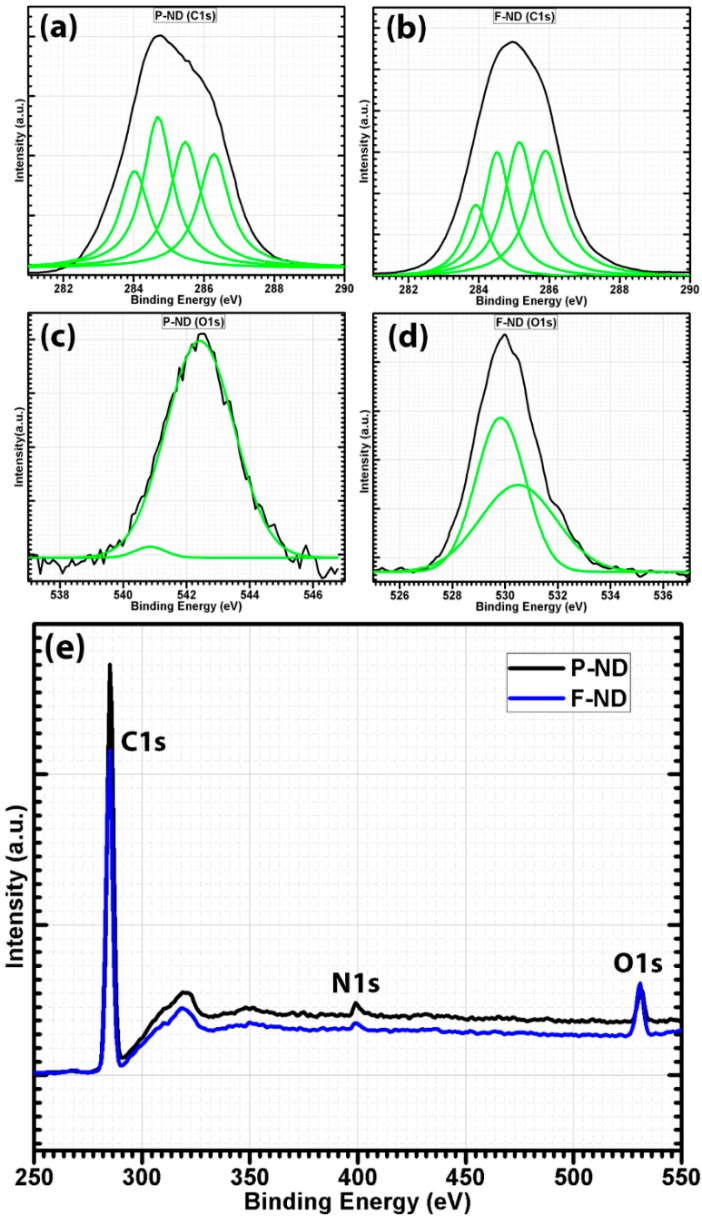
Deconvoluted XPS spectra of C1s (**a**) for P-ND and (**b**) for F-ND; deconvoluted XPS spectra of O1s (**c**) for P-ND and (**d**) for F-ND; (**e**) broad XPS spectra of P-ND and F-ND.

XPS analysis was performed on both P-NDs and F-NDs to further confirm the presence of carboxyl and hydroxyl groups. The deconvoluted C1s core level XPS spectra of P-NDs and F-NDs are shown in Figure 3a,b. It is known that NDs have complex surface structures. Each facet of NDs has its own binding energy. The deconvolution of the P-ND spectrum gives four components with binding energies at about 283.97, 284.6, 285.4, and 286.3 eV whereas the deconvolution of the F-ND spectrum also has four components with binding energies at about 283.92, 284.5, 285.1, and 285.9 eV and these peaks are assigned to C–O, sp^2^ carbon, sp^3^ carbon, and C=O, respectively [28]. The intensity of the bands around 284.5 eV has been decreased in the C1s spectrum of F-NDs as compared to P-NDs. This indicates the removal of sp^2^ carbon content because of functionalization. The band around 285.1 eV also shows a decrement after functionalization which refers to sp^3^ carbon, but the decrease of sp^2^ carbon is more than that of sp^3^ carbon. Furthermore, on comparing both peaks, sp^3^ carbon is still higher than sp^2^ carbon in the C1s spectrum of F-NDs. The elemental ratios obtained during the XPS analysis also emphasized the decrease in carbon content. The elemental ratio of carbon has been decreased from 95 to 91 at %. On the contrary, the strong diamond peak in the XRD gave the clue that this decrease in carbon content is because of the removal of sp^2^ carbon rather than sp^3^ carbon. The bands around 283.9 and 285.9 eV show that oxygen-containing functional groups have been attached on the ND particle surface which is in agreement with the FTIR analysis. The elemental ratio of oxygen has been increased from 3 to 7 at % which further supports the presence of carboxyl and hydroxyl groups. Since the peaks related to oxygen-containing groups are also present in the P-ND C1s spectrum, it can be concluded that they can be part of the impurities embedded in P-NDs [11]. The small elemental ratio of oxygen in P-NDs can be due to the presence of impurities such as non-hydroxyl and non-carboxyl groups embedded in the ND aggregates. However, during functionalization, impurities were removed and additional oxygen-containing functional groups (hydroxyl and carboxyl) were introduced, which can be ascribed to the increase in the elemental ratio of oxygen in F-NDs [11].

**Figure 4:**
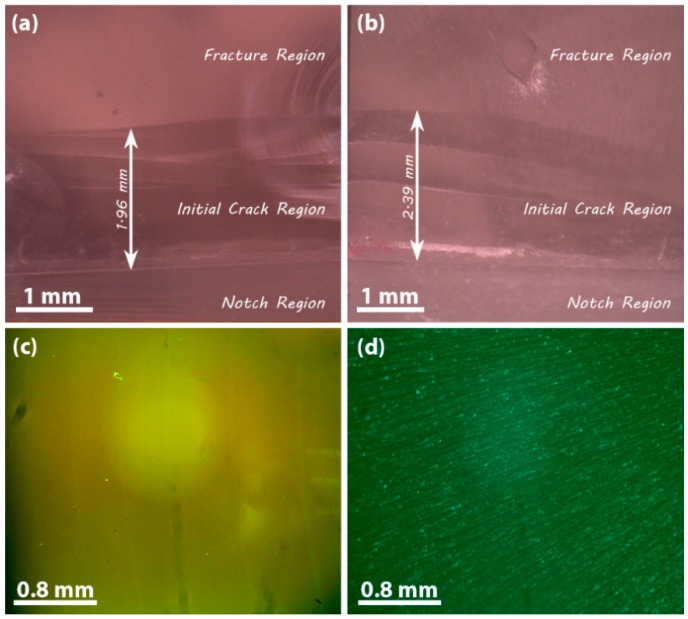
Optical images of (**a**) neat epoxy and (**b**) 10 wt % F-ND/epoxy nanocomposite in dark field to mark the initial crack region and fracture region (direction of crack propagation is from bottom to top); the fracture surfaces of (**c**) neat epoxy and (**d**) 10 wt % F-ND/epoxy nanocomposite in bright field.

On the other side, the O1s spectrum of P-NDs also shows the presence of non-hydroxyl functionalities, as shown in Figure 3c,d. This demonstrates that the oxygen-based impurities were embedded in P-NDs, whereas the bands around 529 eV and 531 eV are attributed to hydroxyl functionalities attached to the NDs’ surface as observed in the O1s spectrum of F-NDs [29,30]. Furthermore, the broad XPS spectra of P-NDs and F-NDs are shown in Figure 3e. On comparing the spectra it is found that the carbon C1s peak is decreased after functionalization whereas the oxygen O1s peak is increased. The decrease in carbon can be attributed to the decrease in sp^2^ carbon after analyzing the deconvoluted peaks of the C1s spectra and XRD spectra. The increase in oxygen can be justified as the attachment of carboxyl and hydroxyl functional groups on the NDs’ surface, in agreement with the deconvoluted O1s spectra and FTIR spectra. So, after these confirmations we have two types of NDs, i.e., those without functional groups (P-ND) and those with functional groups (F-ND). These samples of NDs were used to make a composite with epoxy as a matrix material and their fracture properties were investigated. For fracture testing, nanocomposite samples were prepared according to ASTM D-5045 standards, and to investigate the actual propagation of a crack, an initial crack of about 3 mm was made for each nanocomposite sample by using a sharp razor blade as shown in Figure 4a,b. During the fracture testing, this initial crack propagated in the forward direction due to the applied force and this naturally propagated crack surface was observed via optical microscope. It was observed that the fracture surface of neat epoxy was smooth while the fracture surface of nanocomposite appeared to be rough, as shown in Figure 4c,d. This rough fracture surface is a result of interactions at the interface within the nanocomposite. These interactions at the interface also contributed to higher fracture properties.

**Figure 5:**
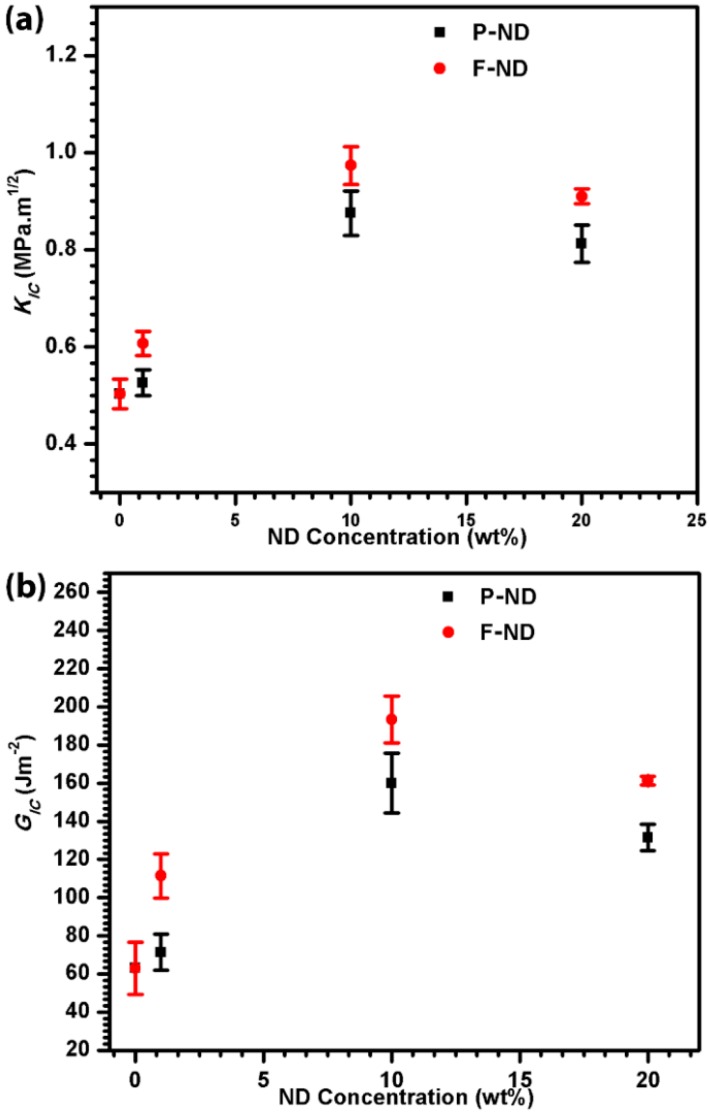
(**a**) Fracture toughness (K*_IC_*) and (**b**) fracture energy (G*_IC_*) of 0, 1, 10, and 20 wt % ND/epoxy nanocomposites.

**Figure 6:**
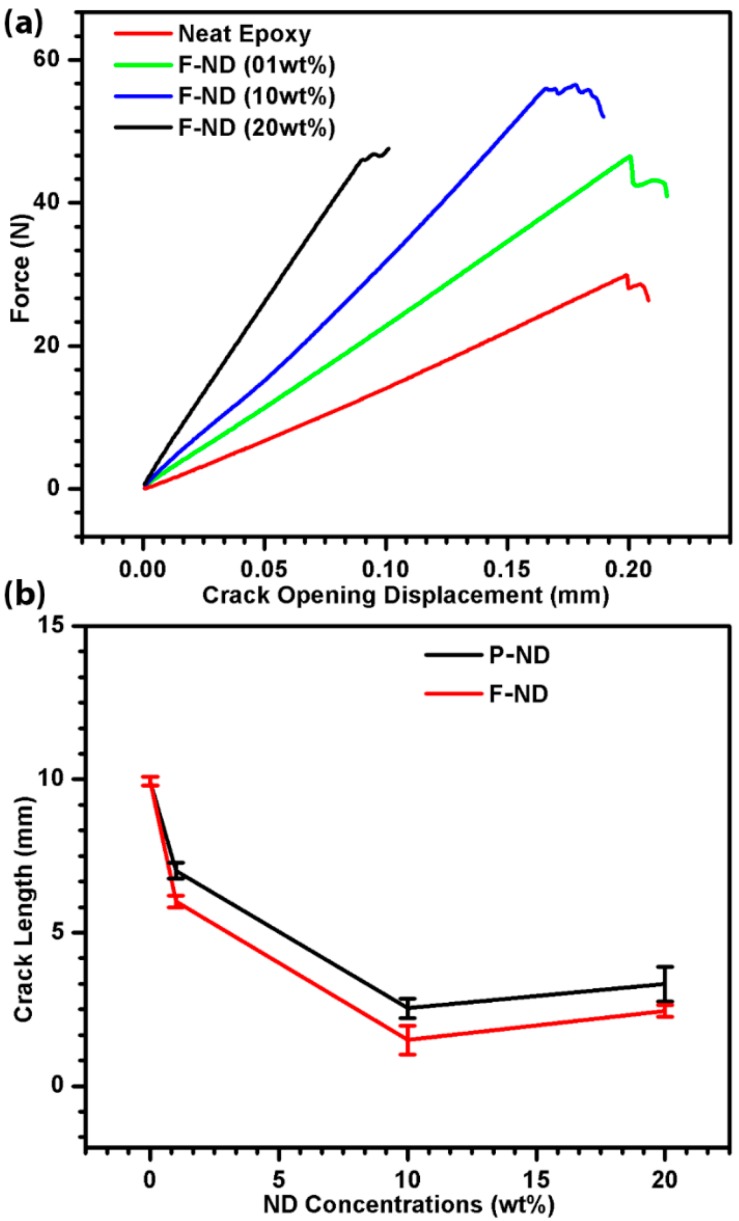
Comparison of (**a**) force-crack opening displacement curves and (**b**) crack lengths of 0, 1, 10, and 20 wt % F-ND/epoxy nanocomposites.

Figure 5a shows the enhancement in fracture toughness with the increase of the ND concentration. The maximum fracture toughness observed was 0.97 MPa·m^1/2^ at 10 wt % F-ND/epoxy nanocomposite, which is twice that of the neat epoxy value. The same trend is observed in fracture energy, as shown in Figure 5b. Again, the maximum fracture energy value is obtained at 10 wt % F-ND/epoxy nanocomposite, which is 193 J·m^−2^. This shows a three-fold increase in the fracture energy whereas Figure 6a is a comparison between the applied force and the crack opening displacement. It shows the inverse relation between the force and crack opening displacement with the increase in ND concentration. It also indicates that nanocomposites with a high concentration of NDs can resist applied force, as a result reducing the crack opening displacement. However, the maximum force they can withstand reduces as the ND content increases, which indicates their brittleness at higher concentrations [11]. Similar findings have been reported in our previous work [11] for tensile properties. Furthermore, Figure 6b shows the comparison between the crack lengths at different concentrations. The minimum crack length observed is 1.5 mm which is for 10 wt % F-ND/epoxy nanocomposite. The fracture toughness, fracture energy and crack length results have shown that 10 wt % is the optimal concentration with maximum gains in our experiments. The rest of the concentrations also show gains in comparison with neat epoxy.

**Figure 7:**
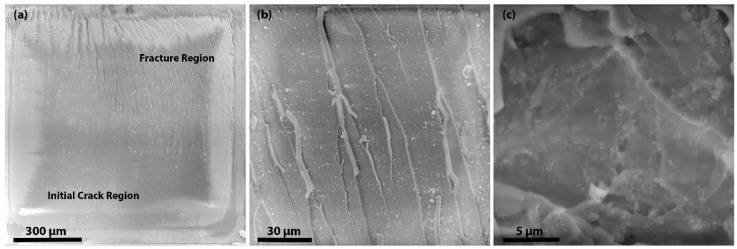
SEM images of the fracture surface for10 wt % of (**a**) P-ND/epoxy and (**b**,**c**) F-ND/epoxy nanocomposites. (Direction of crack propagation is from bottom to top for **a**,**b**).

**Figure 8:**
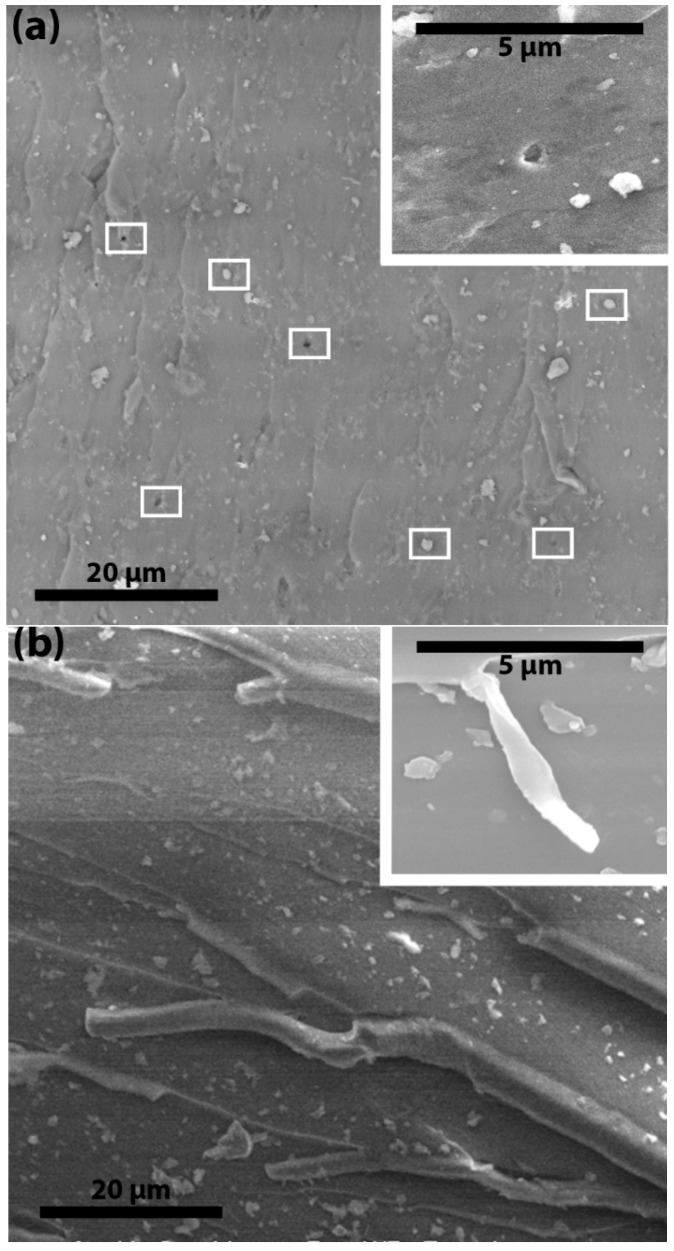
SEM images of the fracture surfaces of 10 wt % (**a**) P-ND/epoxy and (**b**) F-ND/epoxy nanocomposites.

All the values presented in the results are the average of the five best values out of 50 samples for each concentration. The mechanisms responsible for the enhancement in fracture toughness and fracture energy are crack pinning, crack deflection, debonding and plastic void growth. It is known that for crack pinning, the filler size must be greater than the crack opening displacement [31]. As the size of the ND cluster is very small, we suggest that crack pinning has little contribution or effect on the fracture properties of the nanocomposites. To understand the mechanisms, the fracture surfaces were observed by SEM as shown in Figure 7 and Figure 8. In Figure 7a, it is clearly shown that the initial crack region and fracture region have different surface features. Moreover, by observing the fractured surfaces (see Figure 7b), it is clear that crack deflection has a major contribution to higher fracture properties. In Figure 7c, the SEM image of the cross-section of 10 wt % F-ND/epoxy nanocomposite has shown to observe the dispersion of F-NDs inside the epoxy matrix and it is found that the dispersion is almost homogeneous. Furthermore, the surface roughness is direct evidence for crack deflection. Because of crack deflection the crack path length increases which not only increases the surface roughness but also increases the fracture energy. The fracture surface of the F-ND/epoxy nanocomposite is rougher than that of the P-ND/epoxy nanocomposite, as shown in Figure 4c,d and Figure 7b. As we found that the maximum fracture energy is observed in 10 wt % F-ND/epoxy nanocomposite, on the other side, the crack length is the minimum at this concentration. At high concentrations of filler, the distance between the suspended ND particles decreases and the epoxy acts as a glue or adhesive. Because of the uniform distribution (see Figure 7c) of ND particles, the interfaces between the ND particles and the epoxy molecular network overlap by making a web of interfaces [16].

Therefore, at higher concentrations, a small crack opening displacement and small crack length are observed, as shown in Figure 6a,b. In the case of F-ND/epoxy nanocomposites, the interface between the ND particles and the epoxy molecular network is established via covalently attached hydroxyl and carboxyl functional groups. It shows that the interface in the case of F-ND/epoxy nanocomposites is stronger as compared with the P-ND/epoxy nanocomposites where the interface is formed due to adhesion. As suggested, interfaces make a web along with the epoxy molecular network at higher concentrations and this web of interfaces acts as a real constraint for crack propagation. Because of the constraints from the web of interfaces, the crack growth reduces at higher concentrations by losing energy during crack deflection which results in higher values of fracture energy.

Figure 8a shows that the fracture surfaces of P-ND/epoxy nanocomposites have some cavities and semi-naked ND particles (marked by small white rectangles). These cavities or plastic voids were produced because of debonding between the ND particles and the epoxy molecular network. Debonding and plastic void growth are an indication of a weak interface. A weak interface cannot transfer stress from the epoxy network to the ND particles and this failure leads to breakage at the interface. Due to breakage at the interface, particles pull out from the surface and cavities or plastic voids are produced. Sometimes, plastic voids are produced on the opposite surface and semi-naked particles can be seen on the surface under observation. The ratio of the size of the particle cluster and the plastic void gives more information about the debonding. If the plastic void is bigger than the average particle cluster then it indicates debonding within the epoxy network. However, no plastic voids bigger than the average particle cluster are observed in the P-ND/epoxy nanocomposites. On the other side, no plastic voids or semi-naked ND particles are observed in the F-ND/epoxy nanocomposites (see Figure 7b), indicating the strong interface. Sometimes, because of the strong interface, debonding does not occur at the interface but it happens within the epoxy molecular network. Instead of plastic voids, small fibers of epoxy are observed in the F-ND/epoxy nanocomposites, as shown in Figure 8b. Notably, these fiber-like structures clearly indicate that debonding occurred within the epoxy molecular network instead of at the interface by creating a subsequent crack. This subsequent crack travels parallel to the parent crack.

As this subsequent crack does not have sufficient energy, it is blocked by the strong web of interfaces at high concentrations, leaving behind the small nanocomposites fibers. As suggested, because of the strong interface, the crack propagates within the epoxy molecular network and it gets deflected by the ND clusters. Sometimes, instead of deflection, crack splitting may happen. We suggest that crack splitting leads to the fiber-like structure of the epoxy on the surface of the nanocomposites. The plastic void growth, crack deflection and subsequent crack growth effectively limit crack propagation and result in higher values of fracture toughness and energy. Therefore, we suggest that, due to small size, there are more functional groups and these functional groups have a strong interaction with the epoxy molecular network at high concentrations which leads to higher fracture properties and makes the F-NDs a potential candidate for the nanocomposite world.

## 4. Conclusions

In summary, the fracture toughness and fracture energy properties of ND/epoxy nanocomposites are analyzed. A two-fold increase in fracture toughness and a three-fold increase in fracture energy are observed. It is found that F-NDs are a better choice for nanocomposite formation because of the functional groups on their surface. F-ND/epoxy nanocomposites exhibit a rougher surface than that of P-ND/epoxy nanocomposites which indicates the presence of a strong interface. At high concentrations, interfaces overlap by making a web and this web of interfaces stops further crack propagation. In the case of P-ND/epoxy nanocomposites, debonding happened at the interface and plastic voids or semi-naked particles are observed, while debonding for F-ND/epoxy nanocomposites happened within the epoxy molecular network. Strong interface as well as debonding within the epoxy molecular network at high concentrations may lead to subsequent cracks, parallel to the parent crack, via crack splitting, which results in a fiber-like structure on the fracture surface. The plastic void growth, crack deflection and subsequent crack growth are correlated with higher values of fracture toughness and fracture energy.

## Abbreviations

The following abbreviations are used in this manuscript:

ND: Nanodiamond;
P-ND: Pristine Nanodiamond;
F-ND: Functionalized Nanodiamond;
XRD: X-ray Diffraction;
FTIR: Fourier Transform Infrared Spectroscopy;
SEM: Scanning Electron Microscopy;
XPS: X-ray Photoelectron Spectroscopy;
DGEBA: DiGlycidyl Ether of Bisphenol-A;
MHHPA: MethylHexa HydroPhathalic Anhydride;
XANES: X-ray Absorption Near Edge Spectroscopy;
FWHM: Full Width at Half Maximum;
ASTM: American Society for Testing and Materials;
wt %: Weight Percent;
at %: Atomic Percent.
